# Anisotropic thermoelectric behavior in armchair and zigzag mono- and fewlayer MoS_2_ in thermoelectric generator applications

**DOI:** 10.1038/srep13706

**Published:** 2015-09-03

**Authors:** Abbas Arab, Qiliang Li

**Affiliations:** 1 Department of Electrical and Computer Engineering, George Mason University, Fairfax, 22033, US

## Abstract

In this work, we have studied thermoelectric properties of monolayer and fewlayer MoS_2_ in both armchair and zigzag orientations. Density functional theory (DFT) using non-equilibrium Green’s function (NEGF) method has been implemented to calculate the transmission spectra of mono- and fewlayer MoS_2_ in armchair and zigzag directions. Phonon transmission spectra are calculated based on parameterization of Stillinger-Weber potential. Thermoelectric figure of merit, ZT, is calculated using these electronic and phonon transmission spectra. In general, a thermoelectric generator is composed of thermocouples made of both n-type and p-type legs. Based on our calculations, monolayer MoS_2_ in armchair orientation is found to have the highest ZT value for both p-type and n-type legs compared to all other armchair and zigzag structures. We have proposed a thermoelectric generator based on monolayer MoS_2_ in armchair orientation. Moreover, we have studied the effect of various dopant species on thermoelectric current of our proposed generator. Further, we have compared output current of our proposed generator with those of Silicon thin films. Results indicate that thermoelectric current of MoS_2_ armchair monolayer is several orders of magnitude higher than that of Silicon thin films.

The advent of Graphene^1^,^2^,^3^, a two-dimensional (2D) sheet of carbon atoms in honeycomb lattice, has stimulated great interest and intensive research on the properties of 2D materials. More recently, a new family of 2D materials has been proposed, namely Transition Metal Dichalcogenides (TMDs). The presence of a bandgap in some members of TMD family, a crucial property for microelectronics applications, has attracted much attention in comparison with the gapless Graphene. Among all semiconducting TMDs, Molybdenum disulfide (MoS_2_) is the most representative, widely interesting and intensively studied one^4^,^5^,^6^, partially because it is relatively stable and readily available. MoS_2_ has been used as a dry lubricant in automobile industry due to its low friction properties. Recently, it has been studied for applications in field effect transistors^7^,^8^,^9^, photovoltaics^10^ and photocatalysis^11^.

In general, bulk TMDs has a layered structure. Each layer is formed by a plane of transition metal atoms sandwiched between two planes of chalcogen atoms in trigonal prismatic arrangements as illustrated in Fig. 1. Strong intralayer covalent bonding, in contrast to weak interlayer van der Waals forces^12^ make it possible to fabricate high-quality monolayer MoS_2_ by exfoliation technique^12^,^13^,^14^. A desirable bandgap^12^,^13^,^14^,^15^, comparable carrier mobility with those of Si thin film and Graphene nanoribbons^14^,^16^,^17^ together with excellent thermal stability^14^ and surface free from dangling bonds^18^,^19^, makes 2D MoS_2_ a very attractive candidate for device applications^20^,^21^.

Compared to the research progress in its electronic and mechanical characteristics^22^,^23^,^24^, thermoelectric (TE) properties of MoS_2_ have not been widely studied. Thermoelectrics provide a way of converting thermal energy into electricity^25^. Thermoelectric generator is expected to play an important role in increasing demand for clean energy in future^33^. In general, a TE generator module is made of an array of thermocouples. As illustrated in Fig. 2, each thermocouple, the basic unit of a TE generator, is made of a p-type and an n-type semiconductor, named as legs, connected thermally in parallel and electrically in series. Temperature gradient across thermocouple is the driving force inducing electrical current.

The research on thermoelectric materials has been one of the major topics since 1950 s when basic science of thermoelectrics was well founded^26^. Bi_2_Te_3_ and the similar alloys have played a main role in the application of thermoelectric devices. It is well-known that efficiency of thermoelectric conversion can be evaluated by a dimensionless figure of merit 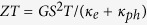, in which 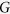, 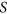, *k*_e_, *k*_*ph*_ and *T* are electrical conductance, Seebeck’s coefficient, electronic contribution to thermal conductance, phonon contribution to thermal conductance and absolute temperature, respectively^26^. In order to have a high ZT, it is desirable to have a high electrical conductance and large Seebeck’s coefficient and low thermal conductance. These parameters mainly depend on the intrinsic properties of materials and they are generally coupled with each other. Enhancement to one of them may degrade the other and the overall effect will not change. In three decades after 1950 s, only incremental progress was made due to the difficulty in fine-tuning of these parameters^27^.

Recently, new wave of research on thermoelectric field has been initiated because nanoscale structures may enhance thermoelectric efficiency. It was shown that quantum confinement of charge carriers in quantum-well super-lattices^28^, quantum-wires^29^ as well as bulk samples containing nanostructured constituents^27^ will enhance thermoelectric conversion. It is known that Density of States (DOS) of low-dimensional materials exhibits sharp changes around Fermi level^27^,^28^,^29^. As a result, Seebeck’s coefficient, which depends on logarithmic derivative of DOS, is significantly enhanced, and hence, the ZT increases. In addition to an increase in Seebeck’s coefficient, low dimensional materials are known to benefit from higher phonon scattering and consequently lower phonon thermal conductance^27^. Low phonon thermal conductivity (*k*_*ph*_) has been already reported for TMDs: 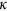
*ph* of MoS_2_ thin films and disordered layered WSe_2_ are about 0.1 W/mK to 1 W/mK^30^ and 0.05 W/mK^31^, respectively. In addition, it has been reported that MoS_2_ has anisotropic thermal properties^32^, which provides another degree of freedom to optimize TE conversion performance. The advantage of nano-scale structures with respect to their bulk counterparts motivates us to study thermoelectric properties of 2D MoS_2_ in both armchair and zigzag orientations.

In this work, thermoelectric properties of mono-, bi-, tri- and quadlayer MoS_2_ in armchair and zigzag directions have been studied for electricity generation. ZT of bulk MoS_2_ has already been reported to be 0.1 at 700 K^33^. In a later study, effect of pressure on thermoelectric properties of MoS_2_ has been investigated^34^. ZT has been reported to increase up to 0.65 in a wide range of pressure and temperature. Thermoelectric performance of monolayer MoS_2_ has been studied and ZT is reported to reach 0.58 in room temperature^35^. In this study, ZT values up to 1.2 in armchair direction has been achieved which is higher than ZT values reported for omnidirectional MoS_2_ structures. Well-established thermoelectric materials include PbTe^36^,^37^ and Bi_2_Te_3_^38^ based alloys, from which higher ZT values around 2.4 have been already achieved at 900 K. However, their substitution with abundant materials is favorable due to scarcity of Te element. This study aims to investigate the possibility of forming high performance thermoelectric generator based on highly available MoS_2_. In addition to abundance of MoS_2_, mono- and fewlayer structures have benefits of possibility of forming high density thermoelectric modules, due to their nano-scale size. In this study, it is found that as the number of layers increases from monolayer to quadlayer, both transmission spectrum and phonon thermal conductance increase. In addition, strong electronic and thermal transport anisotropy is found between zigzag and armchair orientations. Transmission coefficient and phonon thermal conductance of zigzag orientation is higher than those of armchair with the same number of layers. Their effect on ZT has been studied in this work. In addition, TE conversion of Si thin film TE generator with the same thickness as MoS_2_ armchair mono- and fewlayer TE generator has been studied by using Synopsys TCAD software. The comparison indicates that proposed MoS_2_ generator exhibits superior TE conversion efficiency.

## Method

The computational model used in this paper is based on self-consistent density functional theory (DFT) using non-equilibrium Green’s function (NEGF) method^39^,^40^ implemented in QuantumWise ATK software package. Prior to the calculations of carrier transport, the structure has been relaxed to optimized force and stress of 0.05 eV/Å and 0.05 eV/ Å^3^, respectively. The relaxation calculations is implemented by using Generalized Gradient Approximation (GGA) exchange correlation with a Double Zeta Polarized (DZP) basis set and a mesh cut-off energy of 75 Ha.

**Figure 3:**
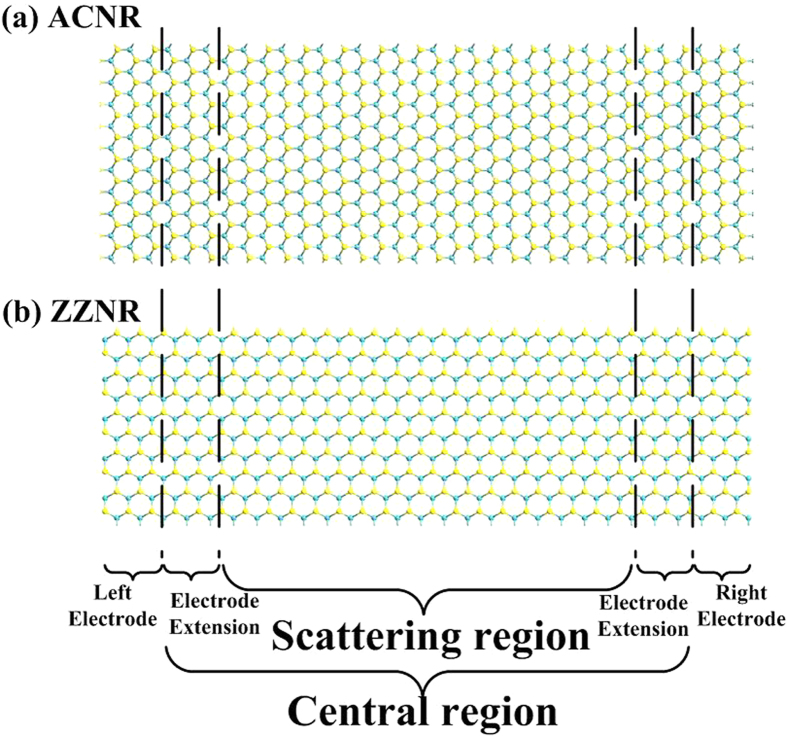
Simulated structures in this paper. Armchair-oriented and zigzag-oriented MoS_2_ are shown in part (**a,b**), respectively. Each device is comprised of three regions: left electrode, central region and right electrode. Central region, itself, contains an extension of electrode regions on both sides and scattering region in the middle. Electrode regions are treated semi-infinitely. Their properties are computed by solving for bulk material. Temperature gradient is biased on electrode regions. Extension of electrode regions in central region, are used to screen out any perturbation introduced in scattering region.

Top view of structures studied in this paper is illustrated in Fig. 3. They can be divided into three regions: left, right and central. Left and right regions are called electrodes, treated with semi-infinite boundary conditions. Their properties can be described by solving for the bulk material. The voltage and temperature bias are applied on electrode regions. Central region includes a repetition of each electrode region in order to screen out perturbations introduced in the scattering regions. In order to have an insight on the effect of lattice orientation and thickness on the intrinsic TE properties of MoS_2_, no perturbation is introduced in the scattering region in calculating ZT.

Central region shown in Fig. 3, should be large enough to accommodate both the voltage and temperature drop within itself. Due to computational constraints, we used 149, 299, 449 and 599 atoms supercell as central region in mono-, bi-, tri- and quadlayer structures, respectively. Using infinitesimal voltage and temperature drop, i.e. working in linear regime, makes our approximation valid. In addition, a vacuum spacing of 20 Å is added to each side of the structure super cell to suppress any interaction caused by periodic boundary condition at out-of-plane direction.

In order to calculate linear transport properties of the system, Landauer-Buttiker^41^ formula is used, in which transport coefficients are calculated from Green’s function. This formulism is correct in absence of inelastic scattering and phase-changing mechanisms. DFT-NEGF method is chosen since it is proven to be a fast and computationally efficient method for a systems containing a large number of atoms^42^,^43^. For DFT calculations, Monkhorst-Pack k-grid^44^ of 1 × 1 × 100 with a density mesh cut-off of 10 Ha is used for supercell within Localized Density Approximation (LDA)^45^ with DZP basis set.

Electrical current 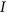 in the linear transport regime is given by:
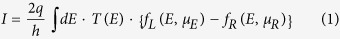
where factor 2 counts for spin degeneracy, *q* is electrical charge of carrier, *h* is Planck’s constant, 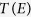 is transmission spectrum coefficient, 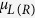 is chemical potential of left (right) electrodes and 
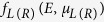
 is the Fermi distribution of left (right) electrode. In linear response regime, it is assumed that 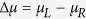 and 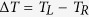 are infinitesimally small. As a result, equation (1) will be reduced to:
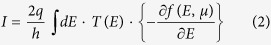


Electronic contribution to TE properties, which is including electrical conductance (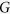), Seebeck’s coefficient (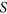) and electronic thermal conductance (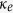), can be calculated by using the followings:
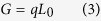

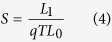

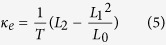
where 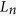 is expressed as:
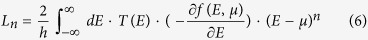


Phonon transmission spectrum is calculated based on parameterization of Stillinger-Weber^46^ potential for MoS_2_^47^ as implemented in Quantum Wise ATK package. Phonon thermal conductance (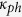) can be calculated as:
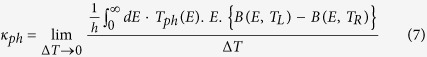
where 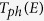 is phonon transmission spectrum; 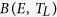 and 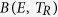 are Bose-Einstein distribution of the left and right electrodes, respectively; 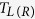 is temperature of left(right) electrode and 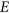 is energy of phonon. In linear response regime, 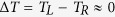 and equation (7) becomes:
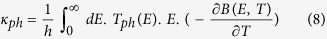


It is worth mentioning that the phonon thermal conductance calculations in this paper are performed in the absence of any phonon decaying mechanisms. Hence, the calculations set the upper limit for phonon thermal conductance of pure MoS_2_. In reality however, there would be a few mechanisms which tend to suppress phonon conduction such as rough surface, edge imperfectness, scattering centers, etc. ZT values calculated in this paper, therefore is the minimum of what actually can be achieved by these materials. TE figure of merit is calculated by using the above information:
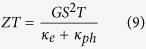


## Results and Discussion

**Figure 4:**
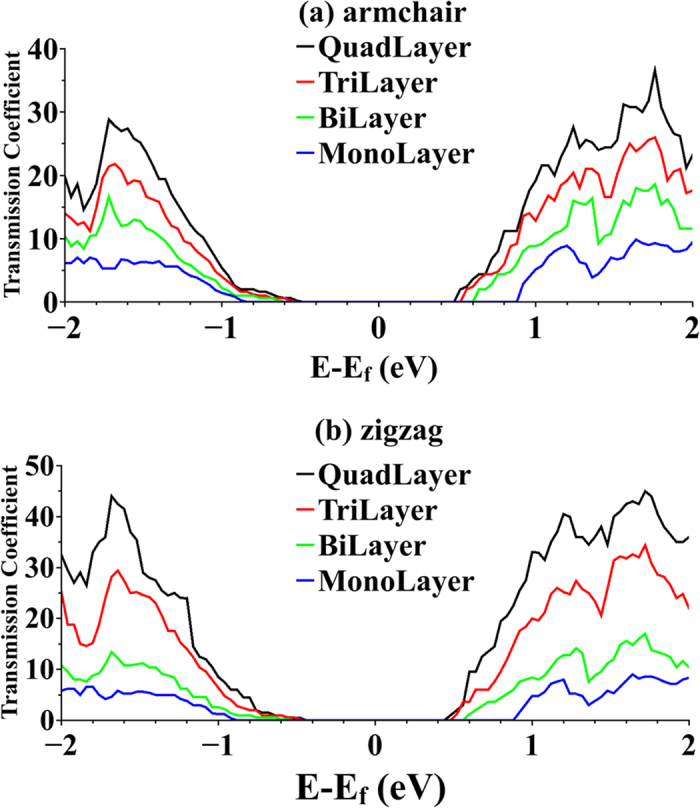
Transmission spectra. Transmission spectrum of (**a**) armchair-oriented and (**b**) zigzag-oriented for mono-, bi-, tri- and quadlayer MoS_2_ calculated based on DFT-NEGF method. Band gap is increasing as number of layers decreases. Transmission coefficients are higher for fewlayer structures, suggesting that each layer provides a conductive channel for carriers.

Transmission spectrum characterizes the electrical behavior of the proposed devices. Electrical factors that affect TE figure of merit include electrical conductance (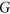), electronic thermal conductance (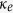) and Seebeck’s coefficient (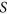). These factors can be derived from transmission spectrum as described in the previous section. Transmission spectrums for monolayer and fewlayer MoS_2_ in armchair and zigzag orientations are illustrated in Fig. 4. Fermi level for is located at 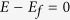. Further study of Fig. 4 indicates that as the number of layers increases from one to four layers, the band gap decreases from 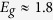
eV to 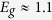
eV which is in good agreement with previously reported values^4^,^5^,^6^. In addition, the amplitude of transmission spectrum coefficient increases as number of layers increases from one to four, indicating that each layer provides an independent channel to conduct carriers^48^. Furthermore, zigzag orientation is found to have higher transmission coefficients in comparison with armchair. It is expected to be more conductive than armchair consequently.

**Figure 5:**
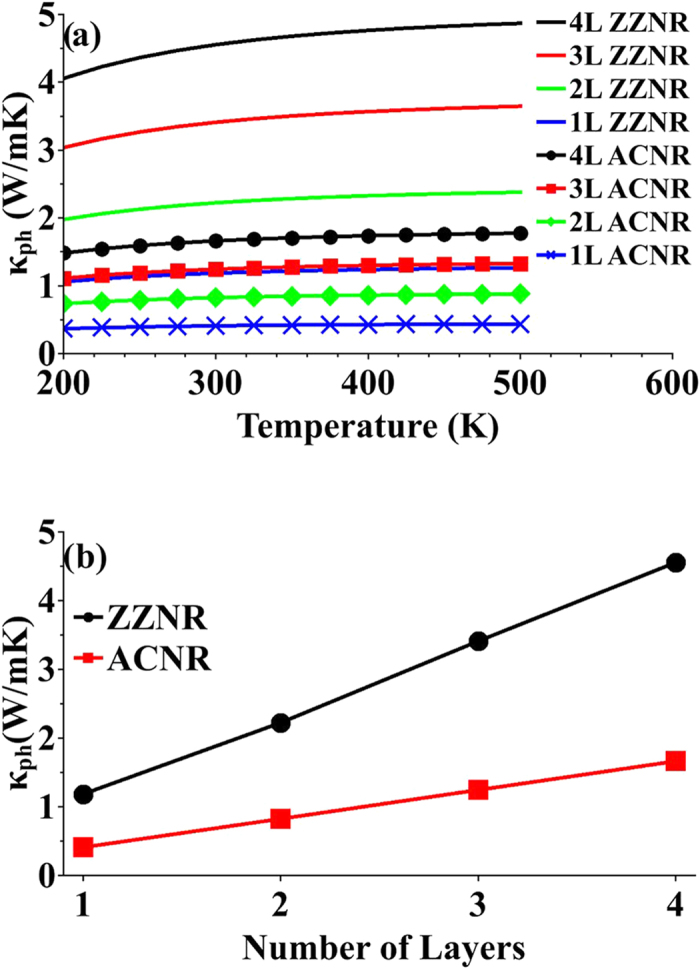
Phonon thermal conductance. (**a**) 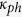 vs. temperature for monolayer and fewlayer armchair- and zigzag-oriented MoS_2_. (**b**) 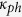 vs. number of layers for armchair- and zigzag-oriented MoS_2_ at *T* = 300k. 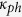 for zigzag orientation shows higher values and greater rate of increase as number of layers increases from monolayer to quadlayer than those for armchair orientation.

In semiconducting materials, phonon thermal conductance (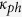) is several times larger than 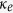 and outplays the impact of 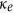 on TE figure of merit. 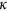
*ph* of monolayer and fewlayer for armchair and zigzag orientations vs. temperature are illustrated in Fig. 5. As shown in Fig. 5, 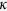
*ph* is almost independent of temperature. It is closely a constant in a wide range of temperatures (from 200 K to 500 K). In addition, zigzag orientation shows larger 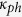 than armchair as was pointed out by Jiang^32^ due to the alignment of one vibrational mode in transport direction along zigzag orientation. These results also suggest that 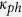 of both zigzag and armchair orientations increases as the number of layers increases. The rate of increase in 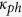 is more in zigzag than in armchair orientation. Our results of 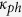 for monolayer MoS_2_ is in a good agreement with findings by Huang^35^.

From factors playing role in TE figure of merit, 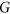 and 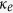 follow the profile of transmission spectrum, i.e. as the Fermi level moves into valence or conduction bands, transmission increases, and hence, there are more carriers to be conducted both thermally and electrically. In contrast to 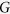 and 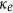, it is typical for semiconductor materials that Seebeck’s coefficient (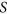) decreases as Fermi level moves into valence and conduction bands. Therefore 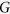 and 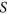 are competing with each other and their product in the form of 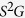, known as power factor, reaches its maximum at an optimum position of Fermi energy^27^,^35^.

**Figure 6:**
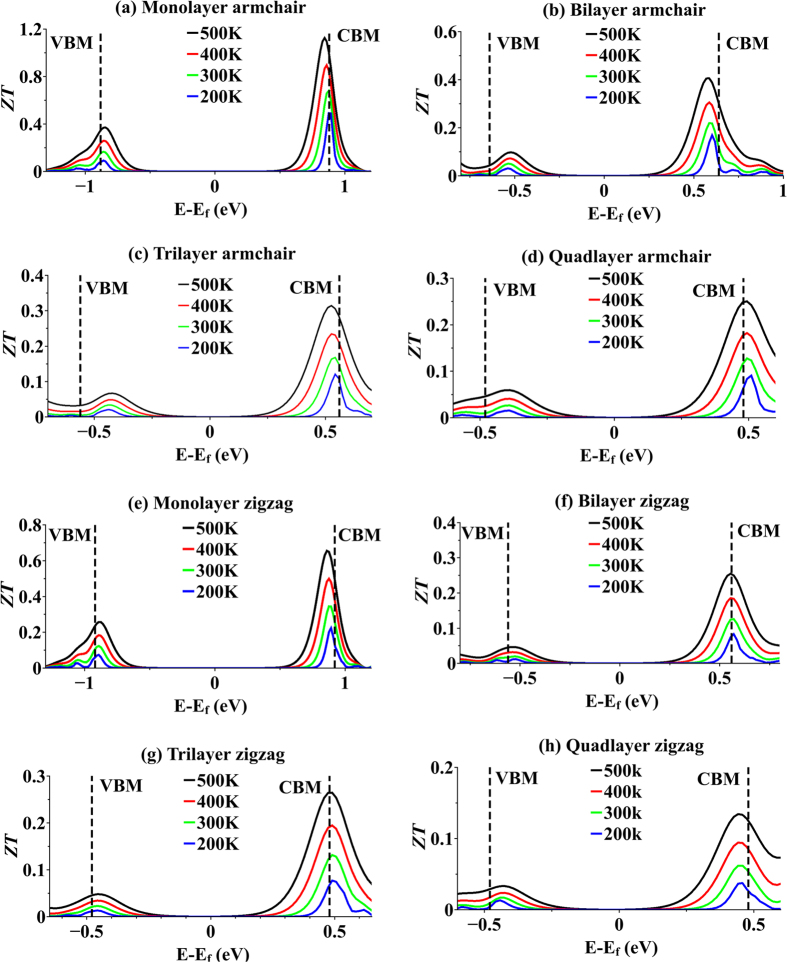
Thermoelectric figure of merit. ZT for monolayer and fewlayer armchair- and zigzag-oriented MoS_2_ vs. Fermi level position for four temperatures. Conduction band minimum (CBM) and valence band maximum (VBM) are shown by vertical dashed lines in each plot.

ZT values of monolayer and fewlayer MoS_2_ in armchair and zigzag orientations vs. Fermi level position at four temperatures are illustrated in Fig. 6. There are two main peaks in ZT, separated by a bandgap, corresponding to valence band and conduction band. Valence band maximum (VBM) and conduction band minimum (CBM) are specified in each plot by vertical dashed lines. In this study, TE figure of merit is referred to as ZT of n-doped or ZT of p-doped as Fermi level is approaching conduction band or valence band, respectively. It is shown in Fig. 6 that for all monolayer and fewlayer structures, ZT values of n-doped are higher than those of p-doped.

As temperature increases, amplitude of ZT also increases since ZT is proportional to the temperature. In addition, rising temperature broadens Fermi distribution. This broadening will populate states in energies higher than Fermi level, which were unpopulated in lower temperatures. These newly occupied states contribute to both electrical and thermal conduction. It means that electrical conductance increases in energies which has insignificant contribution to conduction in lower temperatures, resulting in broadening of ZT peaks vs. energy. Further study of Fig. 6 shows that profile of ZT broadens as number of layers increases for both armchair and zigzag orientations. This behavior can be attributed to the broadening of transmission spectra of both armchair and zigzag orientations as number of layer increases as illustrated in Fig. 4. Despite the increase in transmission coefficients from monolayer to quadlayer, ZT values tend to decrease as number of layers increases. This may be contrary to what is expected. One may expect that higher transmission coefficients is equivalent to more conductivity and hence higher ZT value. The reason for this behavior is due to suppression of out-of-plane vibrational mode in monolayer structures. As it can be seen from Fig. 6, ZT values undergo a sharp drop as structure changes from monolayer to bilayer for both orientations. This drop in ZT value is less pronounced when structure changes from bilayer to quadlayer. In addition, Fig. 6 suggests that ZT value of p-doped structures are smaller than those of n-doped for both orientations. This characteristic can be attributed to lower growth rate in transmission modes as Fermi level moves into valence band compared to when it moves into conduction band as illustrated in Fig. 4.

**Figure 7:**
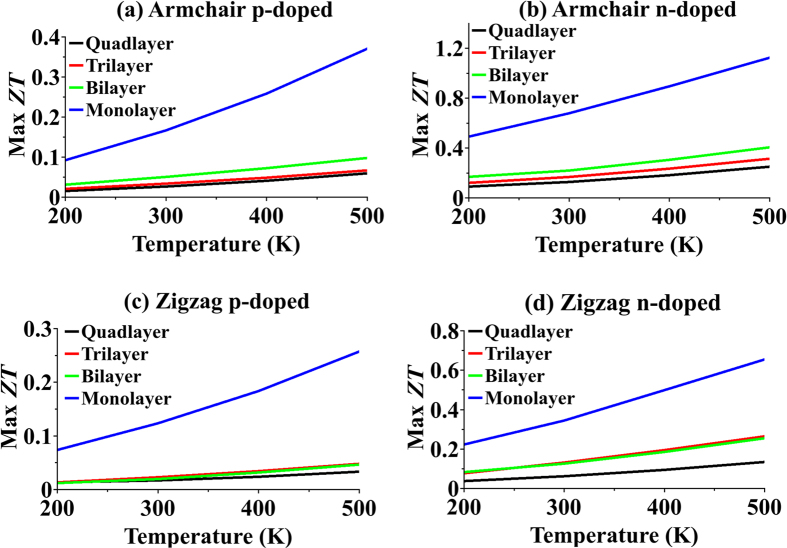
Maximum thermoelectric figure of merit. Maximum ZT for p-doped and n-doped monolayer and fewlayer in both armchair and zigzag orientations vs. temperature.

Peak values of ZT for monolayer and fewlayer armchair and zigzag orientations vs. temperature are shown in Fig. 7. As it was expected from equation (9), ZT is quite linear with temperature. ZT value is monotonously decreasing as number of layers increases. ZT value is larger than unity in n-doped armchair-oriented monolayer at 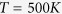. This structure has also the highest p-doped ZT value. Therefore, for both n-type and p-type legs in thermocouple, armchair-oriented monolayer MoS_2_ is the best choice among all structures studied in this paper.

**Figure 8:**
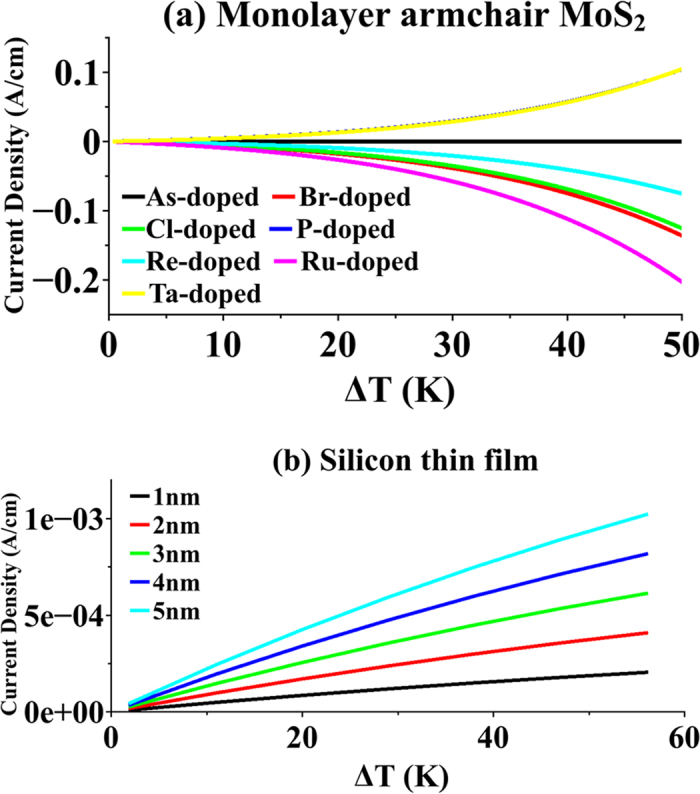
Thermoelectric current of doped armchair-oriented monolayer MoS_2_. (**a**) Thermoelectric current of armchair-oriented monolayer MoS_2_ substitutionally doped with various dopants vs. temperature gradient across it. Transition metal dopants replace Molybdenum and non-metal dopants replace Sulfur. (**b**) Thermoelectric current of Si thin films doped p-type for different film thicknesses vs. temperature gradient across them.

As illustrated in Fig. 6, in order to take advantage of the highest ZT value possible, MoS_2_ should be doped in order to shift Fermi level to the optimum energy of peak value of ZT profile. Substitutional doping of TMD samples has been observed experimentally under exposure to 80 keV electron beam irradiation^49^. Also, a first principal study of effect of this doping approach for transition metal dopants as well as non-metal dopants is reported^50^. In order to examine the TE current of MoS_2_, we have simulated a monolayer MoS_2_ in armchair orientation doped with various dopant species. We followed the same substitutional approach for doping our structure. Transition metal atoms (Re, Ru and Ta) are used as the replacing dopants for Molybdenum, and non-transition metal atoms (As, Br, Cl and P) are used for Sulphur^51^. In order to screen out the perturbation caused by doping properly, only one dopant atom was inserted in central region of device. A temperature gradient has been set across the nanoribbon by fixing the temperature of right electrode to 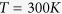 and changing temperature of left electrode from 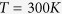 to 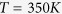 (for device configuration, see Fig. 3). Results are shown in Fig. 8. TE current of monolayer armchair MoS_2_ shows strong dependence on the type of dopant atom. While Arsenic does not show any effect on thermoelectric current, P and Ta showed a similar boost to current. For n-type dopant, Ru exhibits the best current boost in comparison with other dopants. It should be noted that doping in MoS_2_ monolayer at nanoscale will induce device to device performance variation^52^.

These results are compared with TE current of Si thin film doped with acceptor (B) concentration of 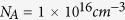 with various film thicknesses (also shown in Fig. 8). For Si thin film with film thickness of 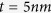, TE current density reaches 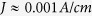 at 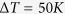. In comparison, monolayer Ru-doped MoS_2_ has TE current density 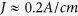 at 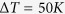, more than two orders of magnitude larger. Decreasing thickness of Si film makes them more resistive and TE current decreases consequently, as shown in Fig.8. Superiority of MoS_2_-based thermocouples will be more dramatic if we compare its TE performance with those of thinner Si films, especially 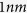-thick Si films which is almost the same thickness of monolayer MoS_2_.

**Figure 9:**
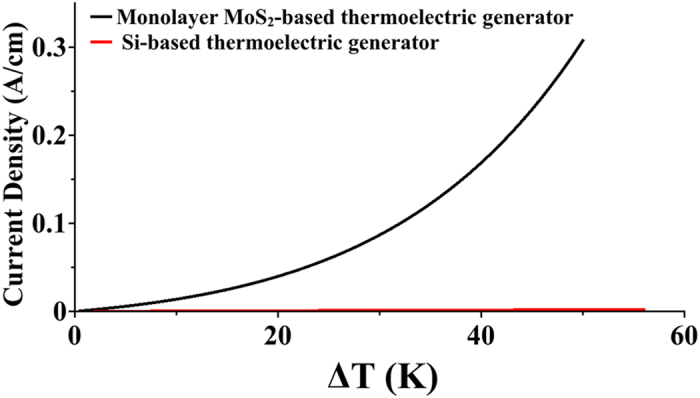
Si thin film vs. armchair-oriented monolayer MoS_2_. Thermoelectric current of thermoelectric generators based on Si thin film in comparison with that of based on doped monolayer MoS_2_.

Thermocouples, as was mentioned in previous section, are made of both p-type and n-type semiconductors. In order to compare the performance of monolayer MoS_2_-based and Si-based thermocouples, TE current of both of these materials is illustrated in Fig. 9. For Si-based thermocouples, p-doped (B) and n-doped (As) films with thickness of 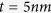 and with doping concentration of 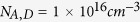 is used. For monolayer MoS_2_ TE conversion, Ru-doped and P-doped are the best n-type and p-type structures, respectively. These two structures are chosen to construct the thermocouple based on monolayer MoS_2_. Fig. 9 shows that thermocouples based on monolayer MoS_2_ are far more superior to thermocouples based on Si thin films.

## Conclusion

In summary, we proposed a TE generator based on monolayer armchair-oriented MoS_2_. In order to find the optimum structure for the proposed thermocouple, first-principle simulation has been performed to calculate TE figure of merit, ZT, for monolayer and fewlayer MoS_2_ in armchair and zigzag orientations. Results indicate that by increasing number of layers, ZT value tends to decrease. This behavior is in contrast to the fact that fewlayer MoS_2_ is more conductive than monolayer in both directions and can be explained by suppression of out-of-plane vibrational modes in monolayer structures. Among all structures studied, monolayer armchair-oriented MoS_2_ is shown to have the highest ZT value as both n-type and p-type semiconducting legs. Moreover, compared to Si thin films, TE current of monolayer MoS_2_ is two orders of magnitude higher.

## Additional Information

**How to cite this article**: Arab, A. and Li, Q. Anisotropic thermoelectric behavior in armchair and zigzag mono- and fewlayer MoS_2_ in thermoelectric generator applications. *Sci. Rep.* **5**, 13706; doi: 10.1038/srep13706 (2015).

## Figures and Tables

**Figure 1:**
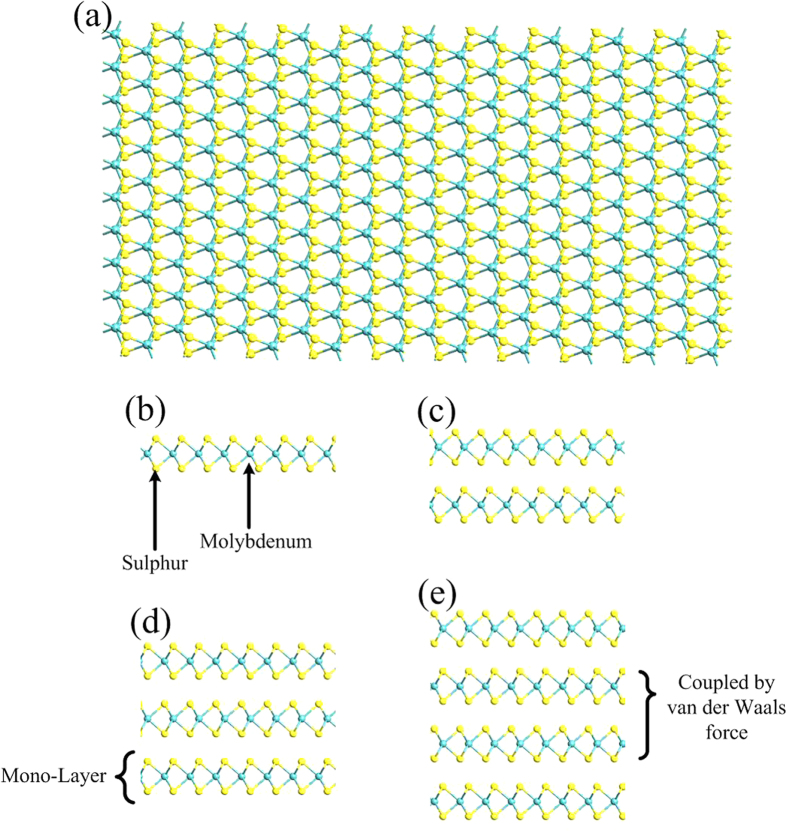
Atomic structure of MoS_2_. (**a**) Monolayer of MoS_2_ is made of a honeycomb sheet of Molybdenum atoms covalently sandwiched between two honeycomb sheets of Sulphur atoms. Bulk of MoS_2_ is formed by monolayers stacked and held on top of each other by van der Waals forces. Side view of mono-, bi-, tri- and quadlayer is illustrated in parts (**b–e**), respectively.

**Figure 2:**
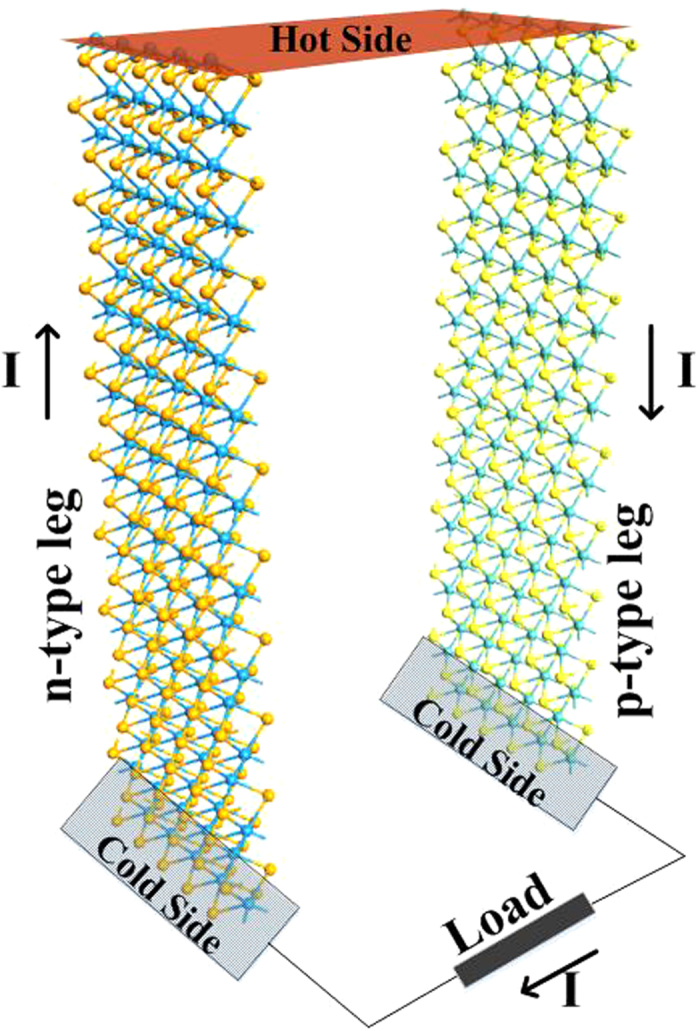
Structure of proposed thermoelectric generator based on monolayer MoS2. It is composed of a p-type and an n-type semiconductor, known as legs. Temperature gradient across thermocouples will induce an electrical current through thermocouple based on thermoelectric phenomenon.

## References

[b1] Novoselov K. S. *et al.* Two-dimensional atomic crystals. Proc. Natl. Acad. Sci. USA. 102, 10451–10453 (2005). 16027370 10.1073/pnas.0502848102 PMC1180777

[b2] Novoselov K. S. A. *et al.* Two-dimensional gas of massless Dirac fermions in graphene. nature 438, 197–200 (2005). 16281030 10.1038/nature04233

[b3] Geim A. K. & Novoselov K. S. The rise of graphene. Nat. Mater. 6, 183–191 (2007). 17330084 10.1038/nmat1849

[b4] Li Y., Zhou Z., Zhang S. & Chen Z. MoS_2_ nanoribbons: High stability and unusual electronic and magnetic properties. J. Am. Chem. Soc. 130, 16739–16744 (2008). 19554733 10.1021/ja805545x

[b5] Ataca C., Sahin H., Akturk E. & Ciraci S. Mechanical and electronic properties of MoS_2_ nanoribbons and their defects. J. Phys. Chem. C 115, 3934–3941 (2011).

[b6] Kou L. *et al.* Tuning magnetism and electronic phase transitions by strain and electric field in zigzag MoS_2_ nanoribbons. J. Phys. Chem. Lett. 3, 2934–2941 (2012). 26292229 10.1021/jz301339e

[b7] Ghatak S., Pal A. N. & Ghosh A. Nature of electronic states in atomically thin MoS_2_ field-effect transistors. Acs Nano 5, 7707–7712 (2011). 21902203 10.1021/nn202852j

[b8] Late D. J., Liu B., Matte H. R., Dravid V. P. & Rao C. N. R. Hysteresis in single-layer MoS_2_ field effect transistors. Acs Nano 6, 5635–5641 (2012). 22577885 10.1021/nn301572c

[b9] Qiu H. *et al.* Electrical characterization of back-gated bi-layer MoS_2_ field-effect transistors and the effect of ambient on their performances. Appl. Phys. Lett. 100, 123104 (2012).

[b10] Gourmelon E. *et al.* MS_2_ (M = W, Mo) photosensitive thin films for solar cells. Sol. Energy Mater. Sol. Cells 46, 115–121 (1997).

[b11] Zong X. *et al.* Enhancement of photocatalytic H_2_ evolution on CdS by loading MoS_2_ as cocatalyst under visible light irradiation. J. Am. Chem. Soc. 130, 7176–7177 (2008). 18473462 10.1021/ja8007825

[b12] Mak K. F., Lee C., Hone J., Shan J. & Heinz T. F. Atomically thin MoS_2_: a new direct-gap semiconductor. Phys. Rev. Lett. 105, 136805 (2010). 21230799 10.1103/PhysRevLett.105.136805

[b13] Splendiani A. *et al.* Emerging photoluminescence in monolayer MoS_2_. Nano Lett. 10, 1271–1275 (2010). 20229981 10.1021/nl903868w

[b14] Radisavljevic B., Radenovic A., Brivio J., Giacometti V. & Kis A. Single-layer MoS_2_ transistors. Nat. Nanotechnol. 6, 147–150 (2011). 21278752 10.1038/nnano.2010.279

[b15] Kam K. K. & Parkinson B. A. Detailed photocurrent spectroscopy of the semiconducting group VIB transition metal dichalcogenides. J. Phys. Chem. 86, 463–467 (1982).

[b16] Liu L., Bala Kumar S., Ouyang Y. & Guo J. Performance limits of monolayer transition metal dichalcogenide transistors. Electron Devices IEEE Trans. On 58, 3042–3047 (2011).

[b17] Yoon Y., Ganapathi K. & Salahuddin S. How good can monolayer MoS_2_ transistors be ? Nano Lett. 11, 3768–3773 (2011). 21790188 10.1021/nl2018178

[b18] Gourmelon E., Bernede J. C., Pouzet J. & Marsillac S. Textured MoS_2_ thin films obtained on tungsten: Electrical properties of the W/MoS_2_ contact. J. Appl. Phys. 87, 1182–1186 (2000).

[b19] Rothschild A., Cohen S. R. & Tenne R. WS_2_ nanotubes as tips in scanning probe microscopy. Appl. Phys. Lett. 75, 4025–4027 (1999).

[b20] Radisavljevic B., Whitwick M. B. & Kis A. Integrated circuits and logic operations based on single-layer MoS_2_. Acs Nano 5, 9934–9938 (2011). 22073905 10.1021/nn203715c

[b21] Wang H. *et al.* Integrated circuits based on bilayer MoS_2_ transistors. Nano Lett. 12, 4674–4680 (2012). 22862813 10.1021/nl302015v

[b22] Yu S., Xiong H. D., Eshun K., Yuan H. & Li Q. Phase transition, effective mass and carrier mobility of MoS_2_ monolayer under tensile strain. Appl. Surf. Sci. 325, 27–32 (2015).

[b23] Kuc A., Zibouche N. & Heine T. Influence of quantum confinement on the electronic structure of the transition metal sulfide TS_2_. Phys. Rev. B 83, 245213 (2011).

[b24] Coehoorn R. *et al.* Electronic structure of MoSe_2_, MoS_2_, and WSe_2_. I. Band-structure calculations and photoelectron spectroscopy. Phys. Rev. B 35, 6195 (1987). 10.1103/physrevb.35.6195 9940850

[b25] DiSalvo F. J. Thermoelectric cooling and power generation. Science 285, 703–706 (1999). 10426986 10.1126/science.285.5428.703

[b26] Goldsmid H. J. in Thermoelectric Refrigeration 1–11 (Springer, 1964).

[b27] Dresselhaus M. S. *et al.* New Directions for Low-Dimensional Thermoelectric Materials. Adv. Mater. 19, 1043–1053 (2007).

[b28] Hicks L. D. & Dresselhaus M. S. Effect of quantum-well structures on the thermoelectric figure of merit. Phys. Rev. B 47, 12727 (1993). 10.1103/physrevb.47.12727 10005469

[b29] Hicks L. D. & Dresselhaus M. S. Thermoelectric figure of merit of a one-dimensional conductor. Phys. Rev. B 47, 16631 (1993). 10.1103/physrevb.47.16631 10006109

[b30] Chiritescu C. *et al.* Ultralow thermal conductivity in disordered, layered WSe_2_ crystals. Science 315, 351–353 (2007). 17170252 10.1126/science.1136494

[b31] Varshney V. *et al.* MD simulations of molybdenum disulphide (MoS_2_): Force-field parameterization and thermal transport behavior. Comput. Mater. Sci. 48, 101–108 (2010).

[b32] Jiang J.-W., Zhuang X. & Rabczuk T. Orientation Dependent Thermal Conductance in Single-Layer MoS2. Sci. Rep. 3, 10.1038/srep02209 (2013). PMC3713516 23860436

[b33] Huai-Hong G., Teng Y., Peng T. & Zhi-Dong Z. Theoretical study of thermoelectric properties of MoS_2_. Chin. Phys. B 23, 017201 (2014).

[b34] Guo H., Yang T., Tao P., Wang Y. & Zhang Z. High pressure effect on structure, electronic structure, and thermoelectric properties of MoS_2_. J. Appl. Phys. 113, 013709 (2013).

[b35] Huang W., Da H. & Liang G. Thermoelectric performance of MX_2_ (M = Mo, W; X = S, Se) monolayers. J. Appl. Phys. 113, 104304 (2013).

[b36] JeffreyáSnyder G. & others. High thermoelectric figure of merit in heavy hole dominated PbTe. Energy Environ. Sci. 4, 2085–2089 (2011).

[b37] Biswas K. *et al.* High-performance bulk thermoelectrics with all-scale hierarchical architectures. Nature 489, 414–418 (2012). 22996556 10.1038/nature11439

[b38] Rosi F. D. Thermoelectricity and thermoelectric power generation. Solid-State Electron. 11, 833–868 (1968).

[b39] Stokbro K., Taylor J., Brandbyge M. & Guo H. in Introducing Molecular Electronics 117–151 (Springer, 2005).

[b40] Brandbyge M., Mozos J.-L., Ordejón P., Taylor J. & Stokbro K. Density-functional method for nonequilibrium electron transport. Phys. Rev. B 65, 165401 (2002).

[b41] Büttiker M., Imry Y., Landauer R. & Pinhas S. Generalized many-channel conductance formula with application to small rings. Phys. Rev. B 31, 6207 (1985). 10.1103/physrevb.31.6207 9935492

[b42] Popov I., Seifert G. & Tománek D. Designing electrical contacts to MoS_2_ monolayers: a computational study. Phys. Rev. Lett. 108, 156802 (2012). 22587274 10.1103/PhysRevLett.108.156802

[b43] Seifert G., Terrones H., Terrones M., Jungnickel G. & Frauenheim T. Structure and electronic properties of MoS_2_ nanotubes. Phys. Rev. Lett. 85, 146 (2000). 10991180 10.1103/PhysRevLett.85.146

[b44] Monkhorst H. J. & Pack J. D. Special points for Brillouin-zone integrations. Phys. Rev. B 13, 5188 (1976).

[b45] Sengupta A. & Mahapatra S. Negative differential resistance and effect of defects and deformations in MoS_2_ armchair nanoribbon metal-oxide-semiconductor field effect transistor. J. Appl. Phys. 114, 194513 (2013).

[b46] Stillinger F. H. & Weber T. A. Computer simulation of local order in condensed phases of silicon. Phys. Rev. B 31, 5262 (1985). 10.1103/physrevb.31.5262 9936488

[b47] Jiang J.-W., Park H. S. & Rabczuk T. Molecular dynamics simulations of single-layer molybdenum disulphide (MoS_2_): Stillinger-Weber parametrization, mechanical properties, and thermal conductivity. J. Appl. Phys. 114, 064307 (2013).

[b48] Das S. & Appenzeller J. Screening and interlayer coupling in multilayer MoS_2_. Phys. Status Solidi RRL-Rapid Res. Lett. 7, 268–273 (2013).

[b49] Komsa H.-P. *et al.* Two-dimensional transition metal dichalcogenides under electron irradiation: defect production and doping. Phys. Rev. Lett. 109, 035503 (2012). 22861869 10.1103/PhysRevLett.109.035503

[b50] Dolui K., Rungger I., Pemmaraju C. D. & Sanvito S. Possible doping strategies for MoS_2_ monolayers: An ab initio study. Phys. Rev. B 88, 075420 (2013).

[b51] Yue Q., Chang S., Qin S. & Li J. Functionalization of monolayer MoS_2_ by substitutional doping: a first-principles study. Phys. Lett. A 377, 1362–1367 (2013).

[b52] Eshun K., Xiong H. D., Yu S. & Li Q. Doping induces large variation in the electrical properties of MoS_2_ monolayers. Solid-State Electron. 106, 44–49 (2015).

